# Benchmarking DFT and Supervised Machine Learning: An Organic Semiconducting Polymer Investigation

**DOI:** 10.1021/acs.jpca.3c04905

**Published:** 2024-01-23

**Authors:** Kyle R. Stoltz, Mario F. Borunda

**Affiliations:** † Physics Department, Oklahoma State University, Stillwater, Oklahoma 74078, United States; ‡ Oklahoma Photovoltaic Research Institute, Stillwater, Oklahoma 74078, United States

## Abstract

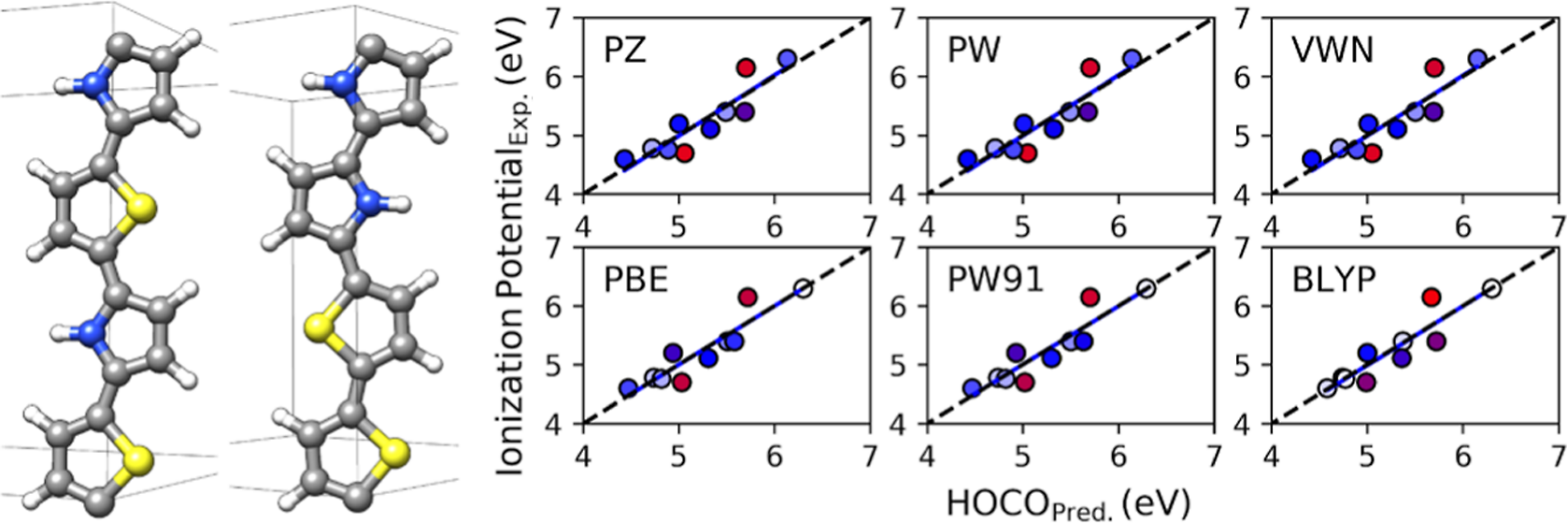

Using a training set consisting of twenty-two well-known semiconducting organic polymers, we studied the ability of a simple linear regression supervised machine learning algorithm to accurately predict the bandgap (BG) and ionization potential (IP) of new polymers. We show that using the PBE or PW91 exchange–correlation functionals and this simple linear regression, calculated BGs and IPs can be obtained with average percent errors of less than 3 and 4%, respectively. We then apply this method to predict the BG and IP of a group of new polymers composed of monomers used in the training set and their derivatives in AABB and ABAB orientations.

## Introduction

As populations grow, so does global energy demand, and one of the most investigated forms of inexpensive and renewable energy is solar. A side effect of power generation with solar cells is the substantial amount of energy lost due to heat dispersion into the atmosphere. Coupling a solar cell with a thermoelectric cell could rectify this problem and increase the overall efficiency. A promising group of materials that can fill both the roles of a solar absorber and be used in thermoelectric generators are semiconducting organic polymers (SOPs). The interest in SOP photovoltaics has increased substantially in recent years with the discovery of nonfullerene acceptor organic solar cells with power conversion efficiencies (PCEs) ranging from 16 to 20%.^1−4^ Also, due to their poor thermal conductivity, SOPs have been studied as promising thermoelectric materials.^5,6^ Unfortunately, these investigations have yielded relatively low figures of merit compared to those of inorganic materials such as BiTe; however, roll-to-roll printing^7−9^ makes them a more viable option. Since monomers can be mixed to form new combinations, many untried possible SOPs could still have a more significant figure of merit.

With just the handful of SOPs investigated in this article, over 400 new combinations of SOPs can be created by mixing any of their monomers in either an ABAB or AABB orientation. Polymers composed of three monomer building blocks in an AABBCC or ABCABC pattern yielded over 8000 new SOPs. Due to the sheer volume of new configurations, experimentally investigating these new SOPs would take significant time and be extremely expensive. Accurate high-throughput screening could accomplish this task and help experimentalists investigate more promising candidates. High-throughput screening has already been successfully applied to molecules,^10^ polymers,^11,12^ searches for novel solar cells,^13,14^ thermoelectrics,^15^ and optoelectronics.^16^ Regardless of whether it is for new solar cells or thermoelectrics, the accuracy of the electronic structure obtained through high-throughput screening is crucial.

**Figure 1:**
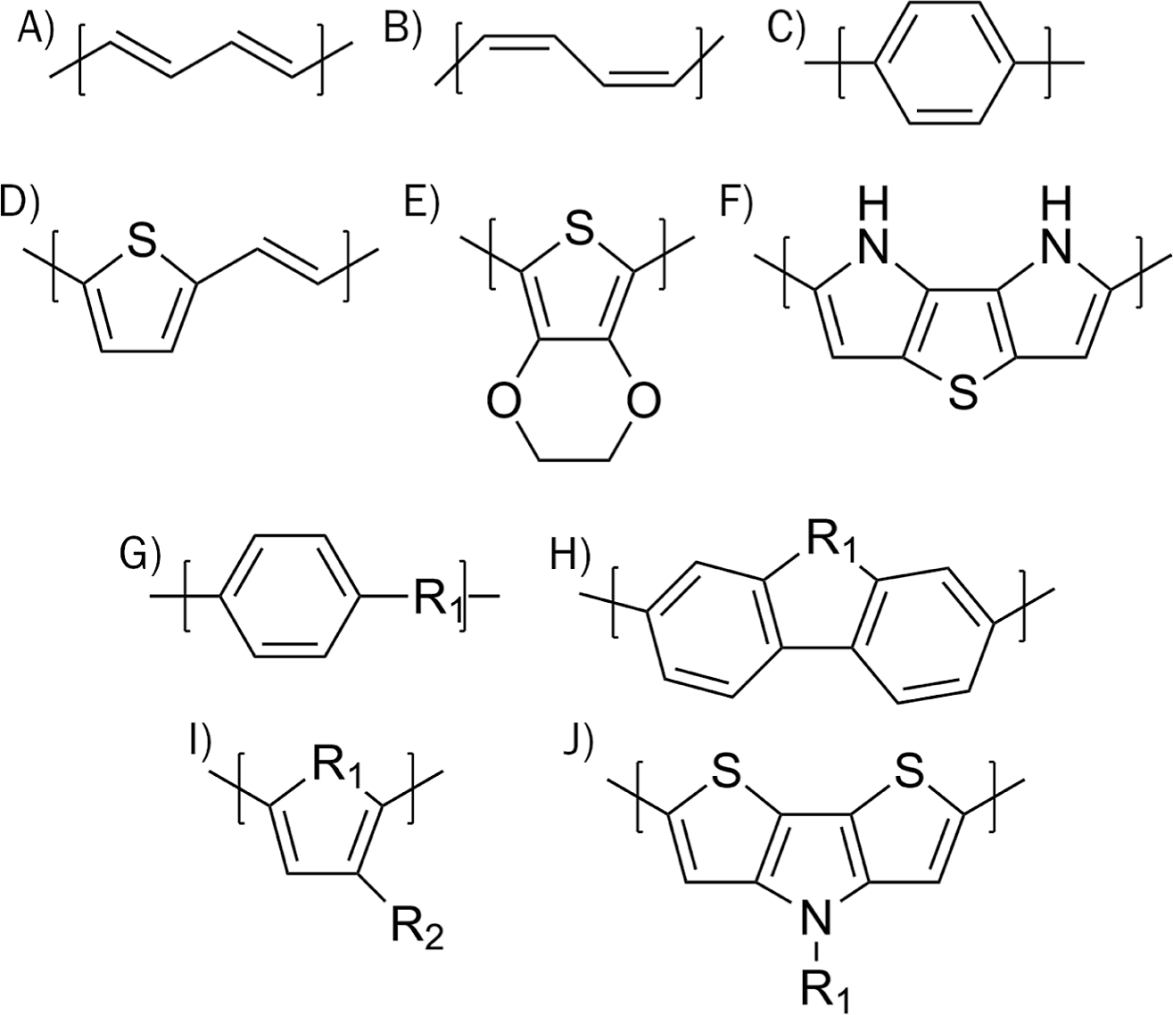
Monomer base structures of the polymers studied (see Table 1).

In this article, we benchmark the computed band gap (BG) and ionization potential (IP) of the SOPs presented in Figure 1 using the VWN,^17^ PZ,^18^ and PW^19^ local-density approximation (LDA) functionals, as well as the PBE,^20^ PW91,^21^ and BLYP^22,23^ gradient-corrected approximation (GGA) *E*_XC_ functionals. From this benchmarking, we obtain a linear calibration function used to correct the calculated BG and IP, a method already shown to work for organic molecules.^24^ Due to their success in accurately describing the BG of semiconductors, the Coulomb hole-screened exchange (COHSEX) and plasmon pole approximation (PPA) *G*_0_*W*_0_ calculations are also applied to PBE-computed systems. Comparing the calibrated BG to the more expensive *G*_0_*W*_0_ calculations, we show that the simple linear correction is more accurate than the *G*_0_*W*_0_ results. We also show that the predictive abilities of these linear corrections through observed training yield BG and IP with relatively small percent errors for the SOPs, thus supporting their use with new combinations of their monomers.

## Computational Methods

For this study, the six *E*_XC_ functionals tested were the VWN,^17^ PZ,^18^ and PW^19^ from the LDA family and the PBE,^20^ PW91,^21^ and BLYP^22,23^ GGA functionals. We utilized norm-conserving pseudopotentials built with the Atomic Pseudopotential Engine code.^25^ The monomers were built and edited in the Avogadro molecular builder tool.^26^ All structure relaxations were carried out with the Quantum Espresso^27^ plane wave software suite, with an energy cutoff of 55 Ry for the wave functions and a force convergence threshold of 0.0103 eV·Å^–1^. The number of Kohn–Sham states we used in all calculations was the number of valence orbitals, plus 20%. A 96-point one-dimensional *k*-space grid was applied along the propagation direction of the polymer chains. Each unit cell contained a dimer aligned along the *Z*-axis with a minimum of 35 Å of vacuum between adjacent polymers. The system was deemed entirely relaxed when the ZZ-component of the stress tensor (ZZ-stress) was below one kbar.

The COHSEX and PPA calculations were done with the YAMBO code^28^ using the relaxed geometry obtained with PBE^20^*E*_XC_. All *G*_0_*W*_0_ were done with a 16-point *k*-space grid and 40 Ry cutoff energy for the wave functions. We used three times the number of valence states for the polarization function and the *G*_0_*W*_0_ summations and a 6 Ry cutoff for the response matrix. To reduce the interactions with adjacent polymer chains, a cylindrical Coulomb potential truncation was applied using the method described by Rozzi et al.^29^

## Polymers Studied and Special Cases

A total of twenty-two SOPs were studied. Their geometries can be seen in Figure 1 with the corresponding atomic substitutions listed in Table 1. They have BGs ranging from 1.64 to 4 eV and IPs from 4.6 to 6.3 eV. The low end of the BG range would result in good solar absorbers, and the energy levels are values needed for achieving reasonable PCEs. The size of the set of polymers used reflects the need for more experimental reports on the BG and IP of polymers. Most polymers were conjugated, meaning they contained alternating single and double bonds between carbon atoms as a backbone, except for poly(phenylene sulfide) (PPS) and polyaniline (PANI). PPS and PANI border on the wide BG semiconductor and comprise a phenylene ring, a sulfur atom for PPS, and a nitrogen and hydrogen atom for PANI. Special care was taken when calculating the final relaxed structures for these polymers since they have an accordion-like structure rather than a straight backbone, as discussed in greater detail below.

**Table 1:** Monomers Studied in This Work

polymer	abbreviation	Figure 1	R1	R2
*trans*-polyacetylene	tPA	(A)		
*cis*-polyacetylene	cPA	(B)		
polyphenylene	PPP	(C)		
poly(thiophene–vinylene)	PTV	(D)		
poly(3,4-ethylenedioxythiophene)	PEDOT	(E)		
poly(1*H*,7*H*-pyrrolo[2′,3′:4,5]-thieno[3,2-*b*]-pyrrole)	PPTP	(F)		
poly(phenylene-sulfide)	PPS	(G)	S	
polyaniline	PANI	(G)	NH	
poly(phenylene–vinylene)	PPV	(G)	HC=CH	
polycarbazole	PCB	(H)	NH	
polyfluorene	PFL	(H)	CH	
poly(9-fluorenone)	P9FL	(H)	CO	
polypyrrole	PPY	(I)	NH	H
polythiophene	PTH	(I)	S	H
polyfuran	PFU	(I)	O	H
poly(3-hexylthiophene)	P3HT	(I)	S	C_6_H_13_
poly(thiophene-3-methanol)	PT3M	(I)	S	CH_2_OH
poly(3-methylthiophene)	P3MT	(I)	S	CH_3_
poly(3-methylpyrrole)	P3MP	(I)	NH	CH_3_
poly(3-octylthiophene)	P3OT	(I)	S	C_8_H_17_
poly(4*H*-dithieno[3,2-*b*;2′,3′-*d*]pyrrole)	PDTP	(J)	H	
poly(poly(4*H*-dithieno[3,2-*b*;2′,3′-*d*]octylpyrrole))	PDTOP	(J)	C_8_H_17_	

### Special Cases

There is a well-known issue with tPA and cPA structural relaxations in density functional theory (DFT). The problem is that the carbons will space themselves so that all the bonds have equal lengths rather than having distinct single and double bonds. This results in an electronic structure with no BGs and thus suggests that tPA is a conductor rather than a semiconductor, which is not the case. To fix this problem, we instead performed a single SCF calculation with the experimental single and double bond lengths of 1.36 and 1.44 Å,^30^ respectively, which opened up a BG in the electronic structure. These experimental bond lengths were also utilized for the cPA single calculation.

**Figure 2:**
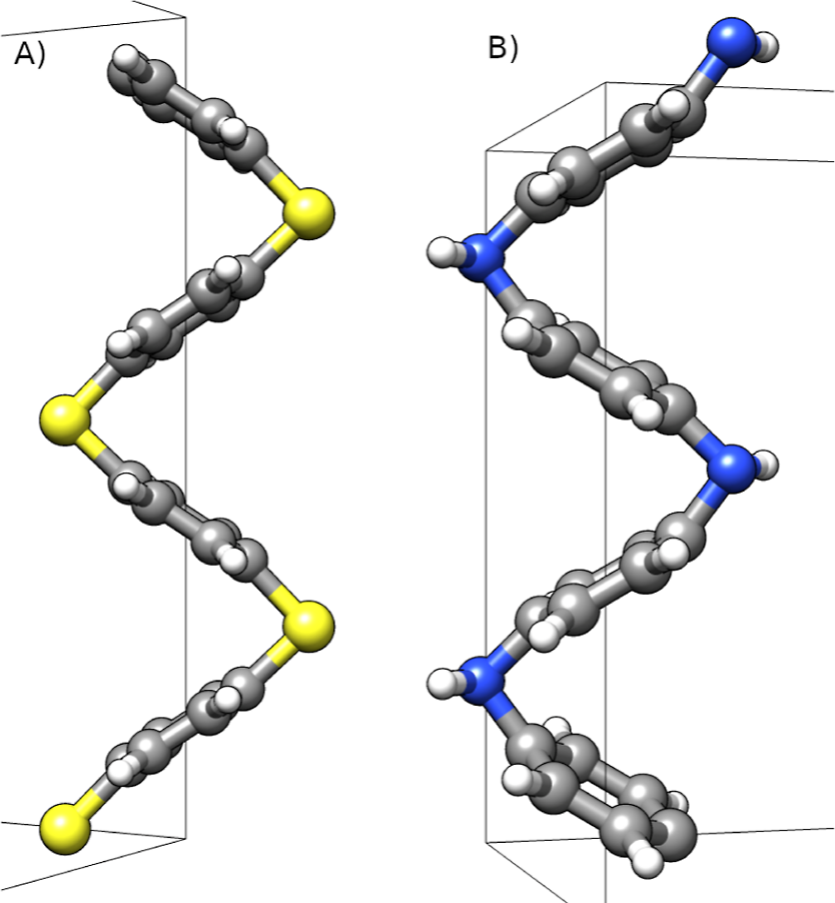
Relaxed PPS and PANI zigzag formation.

**Figure 3:**
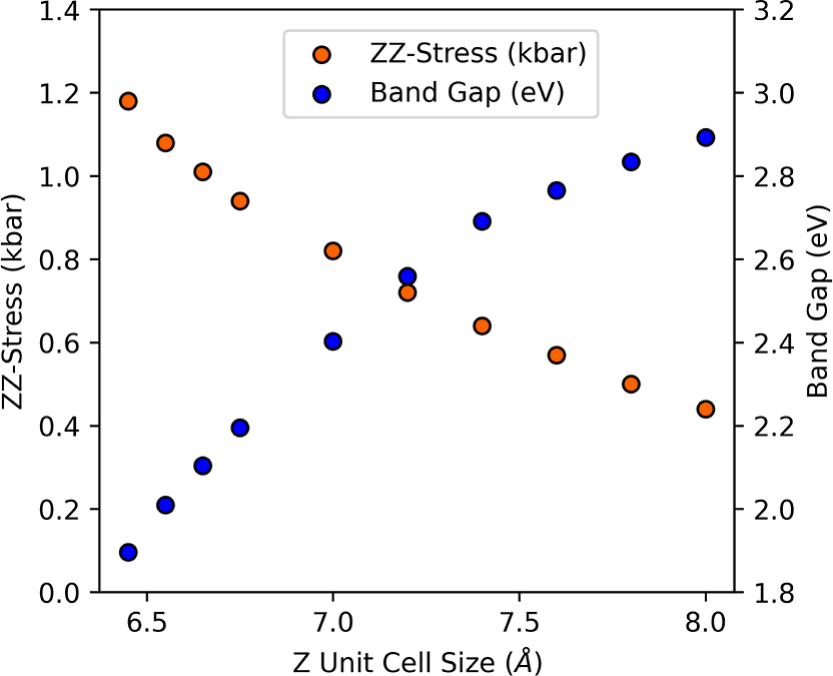
Relaxed PPS BG and ZZ-stress as a function of the *Z*-dimension of the unit cell.

When PPS and PANI were relaxed and their calculated BGs were plotted as a function of the experimental values, they did not align with the conjugated polymers. This is a direct result of the accordion formation that PPS and PANI have when relaxed, as shown in Figure 2. This formation allows the ZZ-stress to fall below 1 kbar, with an overall smaller BG than expected. We performed further relaxations, increasing the dimensions of the unit cell to rectify the problem; the calculated BGs asymptotically approached a value, as shown in Figure 3.

## BG Calibration

The plotted BGs of the relaxed polymers as a function of their experimental values yielded the expected result of the points falling above the one-to-one line. On average, all calculated BGs are underestimated by approximately 50%, but there is a clear linear trend for all six *E*_XC_ functionals (Supporting Information, Figure S1). Given the linear trend, a line of best fit was found where the slope and intercept are used in calibration eq 1, which is then used to correct all calculated values
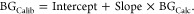
1

**Figure 4:**
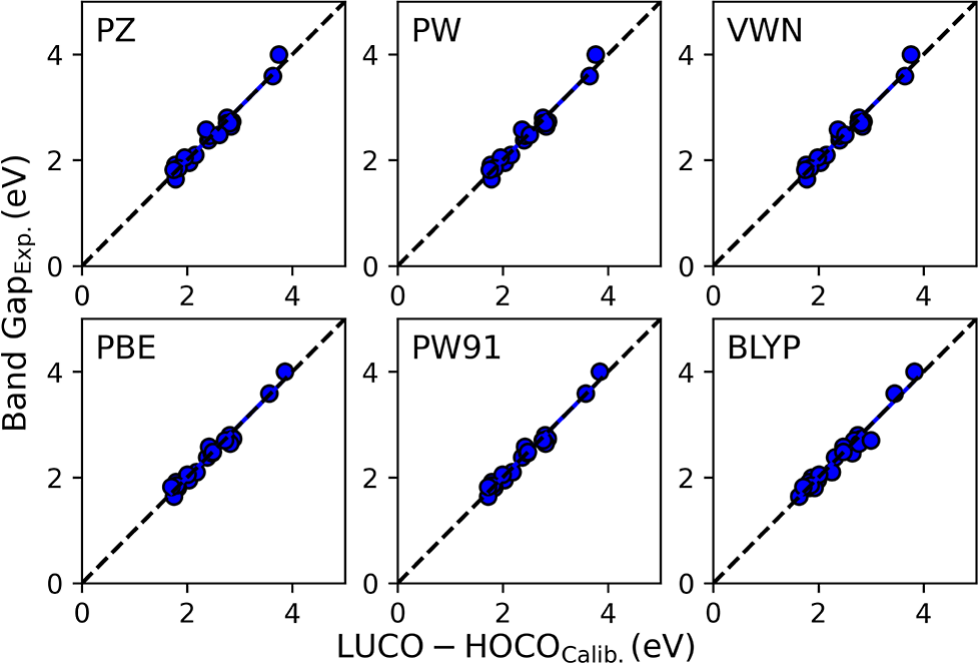
Calibrated BG plotted as a function of the experimental BG.

Once corrected with the calibration function, all data points lie on the one-to-one line or are very close, as shown in Figure 4. We calculated the BGs for each polymer and found their experimental values in the literature. These results are presented in the Supporting Information, Table S1. All functionals performed remarkably well after calibration. We found that the PBE and PW91 GGA functionals perform the best for the set of polymers. The average percent error (A.P.E.) is 2.80% for the PBE and 2.82% for the PW91 *E*_XC_. The BLYP GGA performed worse with an A.P.E. of 3.85%, and the LDA *E*_XC_ performed at 3.36 (PW and VWN) and 3.58% (PZ). These results are encouraging since there is a wide range of BGs, and all *E*_XC_ have A.P.E. less than 4% and an average difference (A.D.) less than 0.1 eV.

**Figure 5:**
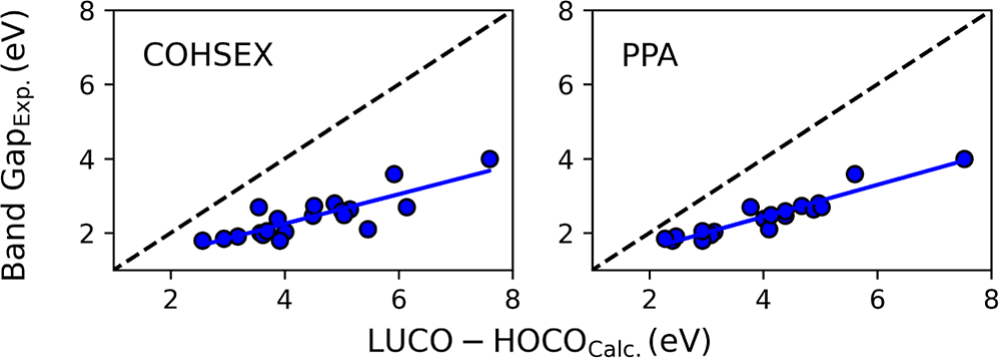
*G*_0_*W*_0_ BG plotted as a function of experimental BG.

The relaxed geometries obtained from the PBE results were used to perform COHSEX and PPA *G*_0_*W*_0_ calculations using the above-mentioned methods to see how the simple linear correction compares to more complex calculations. The results are surprising in that the *G*_0_*W*_0_ calculations yield BGs that are larger than the experimental values. This overestimation is even exaggerated as the measured BG increases, as shown in Figure 5. All of these values are also presented in Table S2 (Supporting Information). Although surprising, the overestimation has been observed before.^31^ However, Ferretti et al.^32^ obtained BGs for tPA and PPV that matched the experimental values very well. One possible explanation for the difference between the Ferretti et al.^32^ results and the ones presented here is the number of valence states used in the calculations. Ferretti et al.^32^ used 288 valence states, where here only three times the number of valence-occupied states were used, which equates to 30 and 114 valence states for tPA and PPV, respectively. Another difference could be that only a single *G*_0_*W*_0_ calculation is performed rather than reaching self-convergence. A single *G*_0_*W*_0_ was performed to compare a single correction to the DFT results and support using the linear calibration function.

## IP Calibration

Similar to the BG calculations, the IPs for all six *E*_XC_ functionals were underestimated, showing the nature of the BG underestimation. Not only are the virtual states underestimated, but so too are the highest occupied states. The underestimation is presented in Figure S2, where the highest occupied crystal orbital (HOCO) is plotted as a function of the experimental IP. All calculated values fall above the one-to-one line. Just like the investigation of the BG, the computed values are corrected with calibration eq 2, with the slopes and intercepts obtained from the linear fits shown in Figure S2.
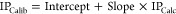
2

**Figure 6:**
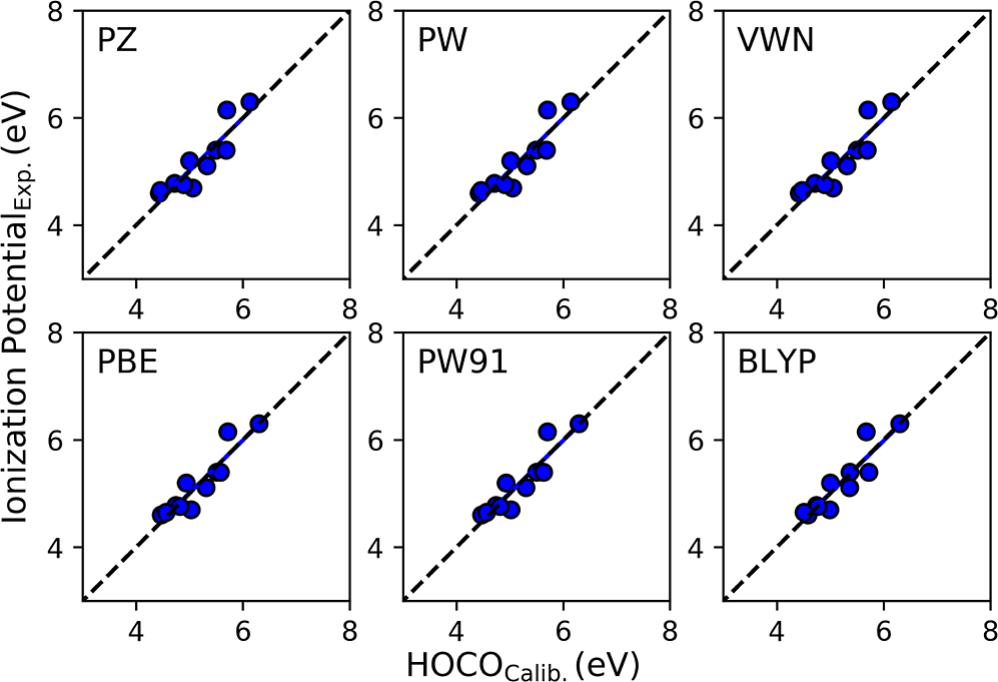
Calibrated IP plotted as a function of the experimental IP.

As shown in Figure 6, the corrected IPs fell either on or close to the one-to-one line. The full results of the calibration are in Table S3 of the Supporting Information. The GGA outperformed LDA functionals. The A.P.E. for BLYP is 2.60, 2.76% for PBE, and 2.79% for the PW91 *E*_XC_, which equates to an A.D. of 0.15 eV or less. The A.P.E. for VWN was 3.31 and 3.34% for both PW and PZ. The A.D. for the LDA functionals was 0.17 eV.

## Accuracy of the Calibrated BG and IP

We estimate the accuracy of the calibration function by using cross-validation. The BG and IP of one of the polymers shown in Figure 1 are obtained from a calibration function obtained from the remaining polymers. For each polymer, we performed cross-validations using all the other polymers as our training database and then predicted the BG and IP of the polymer using calibration equations found from the linear fit of the calculated vs experimental plots of the data set.

**Figure 7:**
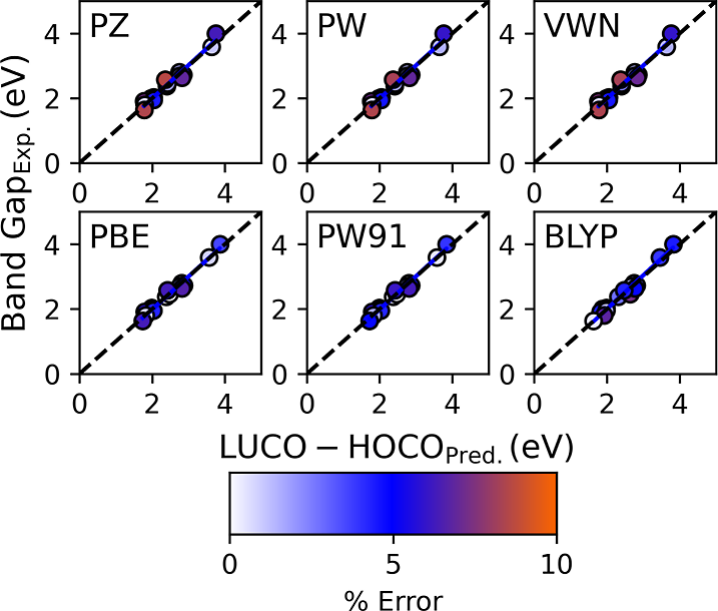
Predicted BG plotted as a function of the experimental BG.

**Figure 8:**
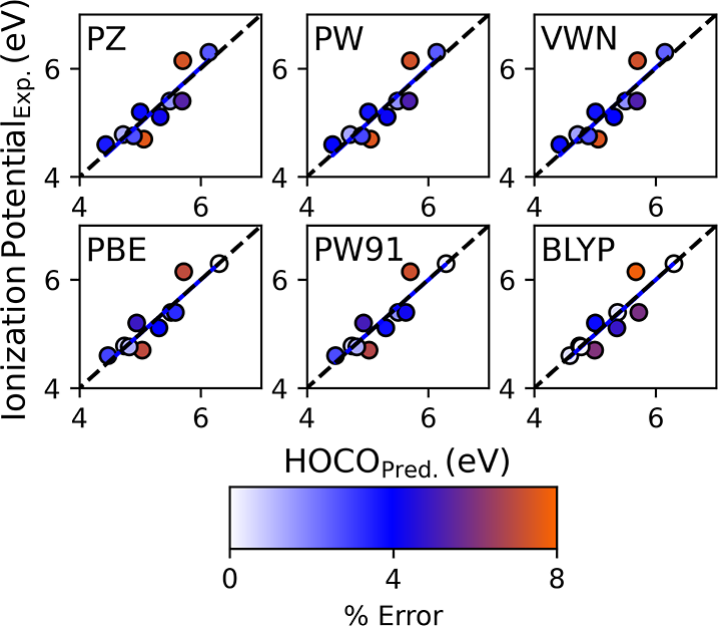
Predicted IP plotted as a function of the experimental IP.

The predicted percent errors for all *E*_XC_ values using this method are presented in Table 2. The predictability for both the BGs and IPs performed reasonably well with all *E*_XC_, having an A.P.E. less than 5%, as presented in Figures 7 and 8. The best performers in the BG predictability study are the GGA *E*_XC_ functionals PBE and PW91, with A.P.E. values of 3.15 and 3.17%, respectively. The BLYP *E*_XC_ was the worst performer at 4.27%, while LDA VWN, PW, and PZ had percent errors of 3.72, 3.73, and 4.01%, respectively. In the IP predictability study, the GGAs outperformed the LDA functionals. The percent error for BLYP was 3.07, 3.22% for PBE, and 3.29% for the PW91 EXC. The IP percent errors for the LDA functionals were 4.00% for VWN, 4.04% for PW, and 4.05% for PZ.

**Table 2:** Predictability Percent Error

functional	BG	IP
PBE	3.15	3.22
PW91	3.17	3.29
BLYP	4.27	3.07
PZ	4.01	4.05
PW	3.73	4.04
VWN	3.72	4.00

Since the overall scope of this project is to generate a method that can predict the BG and IP of new polymers, the methodology must work well with mixtures of the monomers from this study. Since PTV and PPV are a mixture of thiophene and acetylene and phenylene and acetylene monomers in the ABAB formation, they are used to gauge how well this algorithm works for mixtures of monomers. First, looking at the results for PPV, the calculated BG and IP are 2.47 and 5.31 eV for PBE and 2.47 and 5.30 eV for PW91, which matches the experimental BG of 2.46 eV^33^ and is exceptionally close to the 5.11 eV^34^ IP. Now, looking at the *PTV* values, the predicted BG and IP from the PBE supervised training are 1.74 and 4.82 eV, which agree with the experimental 1.64 eV BG and 4.76 eV IP,^34^ and the PW91 results yield a slightly better BG of 1.72 eV and the same IP of 4.82 eV. These encouraging results show that the calibration function method can accurately predict the BGs and IPs of mixed polymers. Unfortunately, the published literature contains only limited experimental measurements of the BGs and IPs, and we could not find results for other possible mixtures.

### Virtual Screening of Mixed SOPs

**Figure 9:**
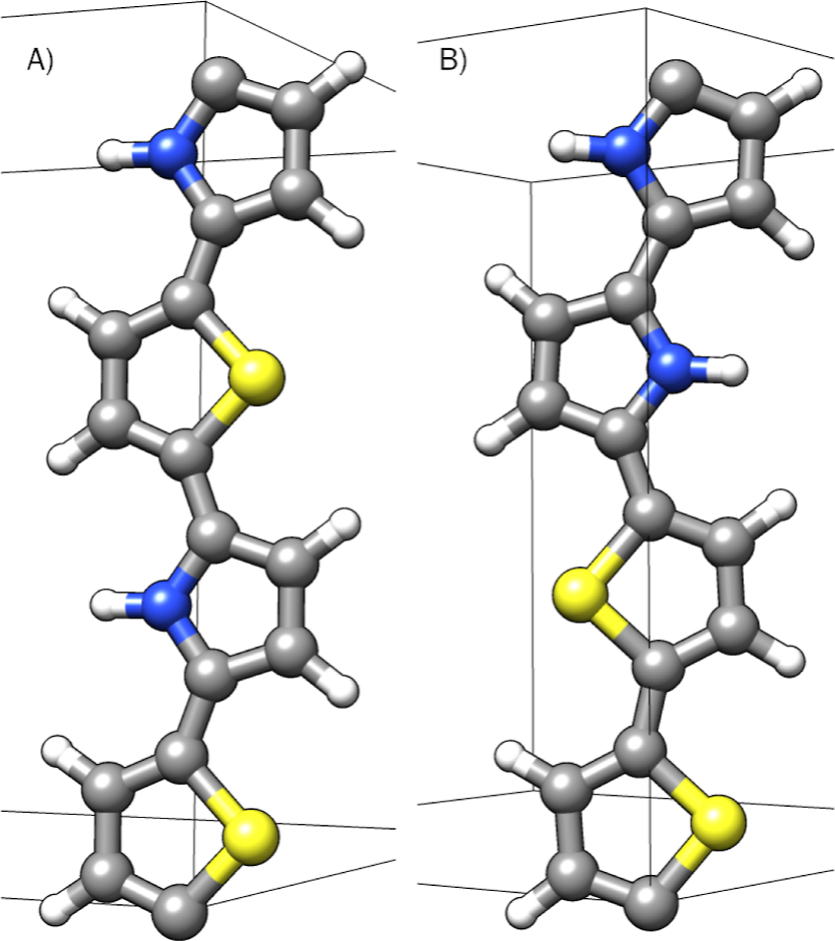
(A) ABAB and (B) AABB orientations of polythiophene/polypyrrole mixtures.

**Figure 10:**
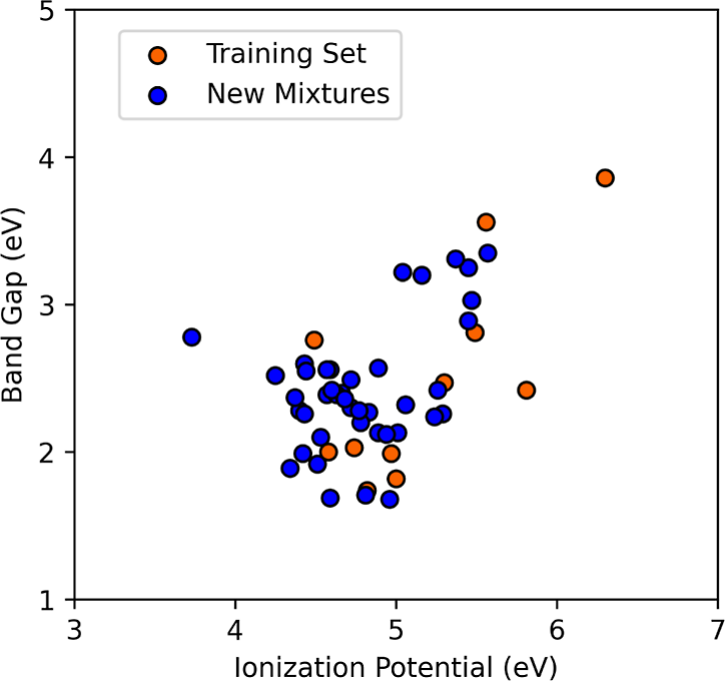
Calibrated BG plotted as a function of the experimental BG.

We have performed a small virtual screening campaign of the copolymers that can be formed by joining two monomers from the training set. These polymers are mixed in the ABAB and AABB orientations, as shown in Figure 9. We then use the calibration scheme to obtain predictions of their BG and IP. We present the results in Figure 10, where the *y*-axis is the BG and the *x*-axis is the IP of possible copolymer mixtures (blue data points) and the training set (in orange). Figure 10 illustrates a wide range of possible BG and IP combos that result from the mixtures. These values fill in the voids left by the initial training set. We also note a trend where most of the new mixtures’ BG and IP values are bunched between 2 and 2.5 eV and between 4.5 and 5 eV, respectively.

Initial findings indicated that the SOPs in the AABB orientation were less likely to relax to a structure with a ZZ-stress of less than 1 kbar. Looking closer, we found this behavior was particularly apparent when the mixed SOP contained PFU and poly(3-methylfuran) (P3MF). Most of the ZZ-stress for the PFU- and P3MF-containing SOPs had considerable negative pressures along the *Z*-direction that never shrank in magnitude as the *Z*-component of the UC was decreased, thus suggesting that these SOPs were unstable. This instability results from the starting angle between the plane of the furan monomer and the monomer with which it is mixed. This starting position might not be correct for new SOPs containing PFU and P3MF monomers; thus, the polymer relaxes to a local minimum rather than a global minimum. The Supporting Information shows this to be the case for the tPA–PFU ABAB and PFU–PPY AABB mixtures. The results in Figure 10 correspond to mixtures where the calculated stress was below 1 kbar; all others were ignored. Tables S6 and S7 present the full results for all mixtures. We discuss this in more detail in the Supporting Information, which also details how the starting angle between the planes of monomers drastically changes the magnitude of the ZZ-stress and how further studies could be done to investigate this aspect.

## Conclusions

This project investigated the ability to correct semiconducting polymers’ calculated BG and IP using three LDA and GGA *E*_XC_ functionals. As expected, all calculated values were underestimated for both BG and IP, but there was a clear linear trend. This led to a linear fit, where the slopes and intercepts were used in a calibration equation that corrected the calculated results. Proving that the calculated BG and IP could be rectified, a supervised machine learning implementation was used to test the predictive ability of the linear fit with the training data. It was found that this simple method could predict the BG and IP with an A.P.E. of less than 5%. The final test was to examine how accurately the BG and IP were predicted for PTV and PPV since they are a mixture of two monomers. It was demonstrated that the supervised machine learning method with the training data gave accurate results.

With the accuracy of our method demonstrated, we then moved on to find the BG and IP of 37 new SOPs by taking 8 SOPs from our training set and mixing them two at a time in an ABAB or AABB orientation. Finding optimal organic materials in solar cells continues to be a goal of the organic semiconductor community.^1−4^ A recent study showed that the difference between the IP of the donor and acceptor should range between 0.4 and 0.65 eV.^35^ The methodology presented in this paper will help screen SOPs with varying BGs with IPs that fall within that specified range. We found new SOPs with new BGs and IPs ranging from 1.66 to 3.36 and 3.73 to 5.57 eV, respectively. Another contribution from this study to organic solar cell research is the calculated BG of the PFU and tPA mixtures in the ABAB orientation. With a value of 1.69 eV, it is the smallest value found from the new mixtures and provides the SOP whose absorption spectra could align closer to the peak of the solar spectrum. These varieties of predicted results should be of interest to the organic electronics community.

## Data Availability

All calculations used the Quantum Espresso^27^ or Yambo^28^ codes. The input files for both codes, including the Cartesian coordinates of all the polymers discussed in this paper, are available in the Supporting Information as a ZIP file. The molecular graphics were performed with UCSF Chimera, developed by the Resource for Biocomputing, Visualization, and Informatics at the University of California, San Francisco, and are available at: http://www.cgl.ucsf.edu/chimera/.^36^

## Supporting Information Available

The Supporting Information contains the Supporting Information is available free of charge at https://pubs.acs.org/doi/10.1021/acs.jpca.3c04905.

Calculated BGs and IPs versus their experimental values and calibrated results (PDF)
