# Supplementary material for: Benchmarking DFT and Supervised Machine Learning: An Organic Semiconducting Polymer Investigation
Source: J Phys Chem A. 2024 Jan 23;128(4):709–15. doi: 10.1021/acs.jpca.3c04905 (PMC10839824; doi:10.1021/acs.jpca.3c04905)
Supplement: Supplementary file 1 — jp3c04905_si_001.pdf [file jp3c04905_si_001.pdf]

Supporting Information: Benchmarking DFT and Supervised Machine Learning: An Organic Semiconducting Polymer Investigation

Kyle R. Stoltz† and Mario F. Borunda*,†,‡

†*Physics Department, Oklahoma State University, Stillwater, Oklahoma 74078, USA*

‡*Oklahoma Photovoltaic Research Institute, Oklahoma 74078, USA*

E-mail: mario.borunda@okstate.edu

Band Gap Study

In Fig. S1, we present the calculated band gaps (BG) plotted as against the experimental values reported in the literature for the polymers presented in the manuscript. We list the slope and intercept of the line of best fit and the R^2 value of the fit. The BGs are then calibrated as shown in the manuscript. Table S1 presents the corrected calculations and experimentally measured values for the polymers.

Table S2 presents the COHSEX and PPA G_0W_0 calculations and experimentally measured values for the polymers.

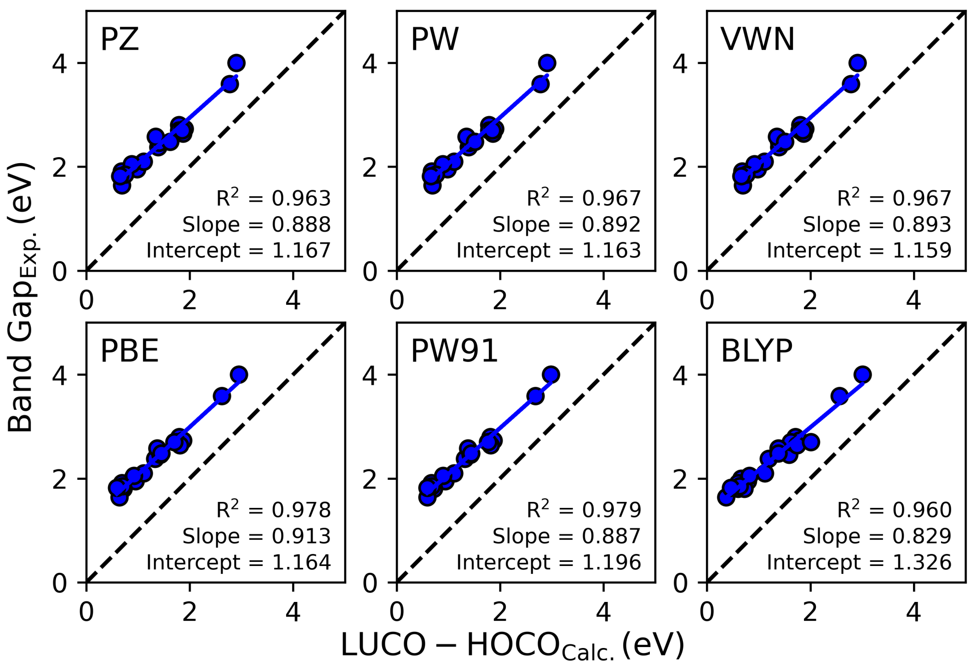

Figure S1: The calculated BG, obtained from the difference between the lowest unoccupied crystal orbital (LUCO) and the highest occupied crystal orbital (HOCO), plotted as a function of the experimental BG.

Table S1: Calibrated Band Gaps in eV for each polymer. The average percent error (A.P.E.) and the average difference (A.D.) are also listed for each functional.

Polymer	VWN	PW	PZ	PBE	PW91	BLYP	Exp
PPP	2.76	2.76	2.75	2.81	2.80	2.74	2.80[S1]
PTH	2.05	2.05	2.04	1.99	1.99	1.98	2.03[S2]
PFU	2.40	2.41	2.40	2.38	2.37	2.31	2.38[S3]
PPV	2.40	2.42	2.42	2.47	2.47	2.64	2.46[S4]
PCB	2.86	2.86	2.85	2.87	2.86	2.81	2.73[S5]
PPY	2.76	2.76	2.76	2.76	2.75	2.67	2.70[S6]
P3HT	1.98	1.98	1.97	2.00	2.00	1.87	2.09[S7]
PEDOT	1.80	1.79	1.79	1.81	1.82	1.82	1.80[S8]
PT3M	1.76	1.77	1.77	1.79	1.79	1.82	1.91[S9]
PANI	3.64	3.64	3.63	3.56	3.57	3.45	3.59[S10]
PFL	2.83	2.82	2.82	2.82	2.81	2.77	2.64[S11]
P9FL	2.37	2.37	2.36	2.42	2.42	2.47	2.58[S12]
P3MT	2.04	2.04	2.04	2.03	2.03	1.98	1.95[S13]
tPA	1.78	1.79	1.79	1.82	1.83	1.92	1.80[S14]
PPS	3.76	3.76	3.75	3.86	3.84	3.82	4.00[S15]
PTV	1.78	1.78	1.78	1.74	1.72	1.63	1.64[S16]
cPA	2.15	2.15	2.15	2.18	2.18	2.26	2.10[S17]
P3MP	2.81	2.81	2.81	2.71	2.77	3.00	2.70[S18]
P3OT	1.98	1.96	1.95	2.00	1.99	2.01	2.05[S19]
PDTP	1.84	1.84	1.83	1.80	1.80	1.85	1.85[S20]
PPTP	2.51	2.51	2.60	2.49	2.47	2.48	2.48[S20]
PDTOP	1.75	1.75	1.75	1.71	1.72	1.82	1.82[S20]
A.P.E.	3.36	3.36	3.58	2.80	2.82	3.85	
A.D. (eV)	0.08	0.08	0.09	0.06	0.07	0.09	

Table S2: G_0W_0 Band Gaps (eV)

Polymer	PBE-Calib	COHSEX	PPA	Exp
PPP	2.81	4.88	4.97	2.80[S1]
PTH	1.99	4.01	3.14	2.03[S2]
PFU	2.38	3.87	4.01	2.38[S3]
PPV	2.47	4.49	4.38	2.46[S4]
PCB	2.87	4.52	4.67	2.73[S5]
PPY	2.76	3.55	3.77	2.70[S6]
P3HT	2.00	3.57	2.94	2.09[S7]
PEDOT	1.81	2.56	2.40	1.80[S8]
PT3M	1.79	3.18	2.46	1.91[S9]
PANI	3.56	5.92	5.60	3.59[S10]
PFL	2.82	5.14	4.88	2.64[S11]
P9FL	2.42	5.01	4.38	2.58[S12]
P3MT	2.03	3.62	3.08	1.95[S13]
tPA	1.82	3.91	2.93	1.80[S14]
PPS	3.86	7.59	7.53	4.00[S15]
cPA	2.18	5.46	4.10	2.10[S17]
P3MP	2.71	6.14	5.02	2.70[S18]
P3OT	2.00	3.69	2.93	2.05[S19]
PDTP	1.80	2.93	2.27	1.85[S20]
PPTP	2.49	5.04	4.13	2.48[S20]

IP Calibration

Figure S2 presents our calculated ionization potentials (IPs) against the experimental IP. We list the slope and intercept of the line of best fit and the R^2 value of the fit. The IPs are then calibrated as shown in the manuscript. Table S1 presents the corrected calculations and experimentally measured values for the polymers.

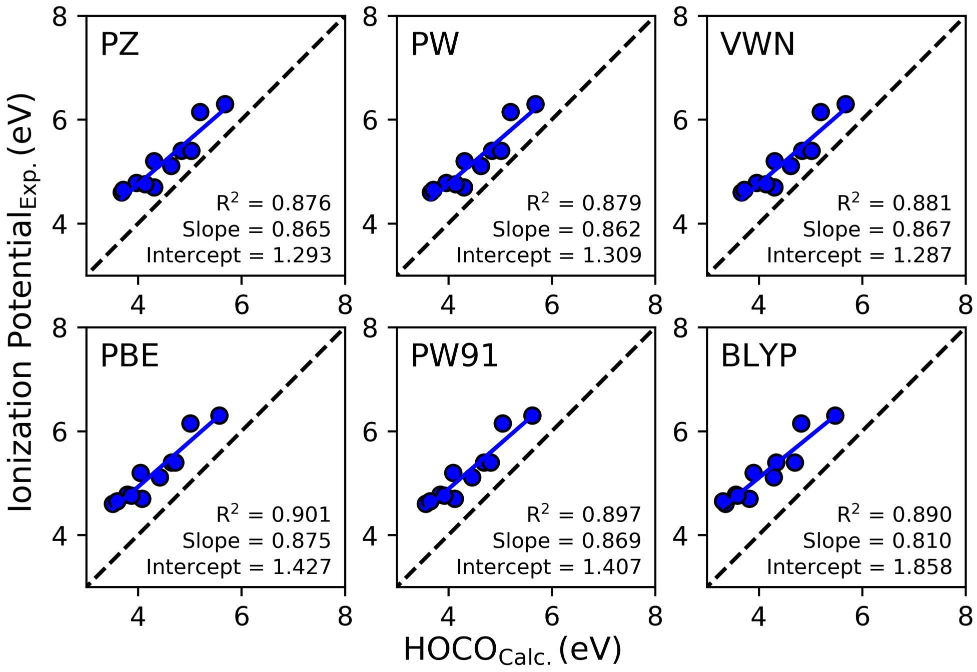

Figure S2: The calculated IP plotted as a function of the experimental IP.

Stress Study

As alluded to in the manuscript, the starting angle between the planes of the monomers can affect the Z-component of the stress tensor of the relaxed polymer. We combined PFU and tPA in the ABAB orientation to test this idea. We ran multiple relaxations following the process outlined in the "Computational Methods" section with different starting angles between the planes of the two monomers. It is worth noting that this was the only new SOP

Table S3: Calibrated Ionization Potential (eV)

Polymer	VWN	PW	PZ	PBE	PW91	BLYP	Exp
tPA	5.02	5.02	5.02	5.00	4.99	4.96	4.70[S21]
PPP	5.48	5.48	5.48	5.49	5.48	5.38	5.40[S22]
PPS	6.21	6.21	6.21	6.30	6.29	6.30	6.30[S23]
PPY	4.47	4.47	4.47	4.49	4.50	4.58	4.60[S6]
PPV	5.29	5.31	5.31	5.30	5.29	5.34	5.11[S16]
PAN	5.64	5.64	5.64	5.56	5.60	5.66	5.40[S24]
PTH	5.02	5.02	5.02	4.97	4.97	5.02	5.20[S16]
P3MT	4.72	4.73	4.72	4.74	4.74	4.74	4.78[S25]
P9FL	5.80	5.79	5.79	5.81	5.80	5.76	6.15[S26]
PTV	4.88	4.88	4.88	4.82	4.81	4.76	4.76[S16]
P3HT	4.51	4.51	4.51	4.58	4.58	4.54	4.65[S27]
Average Percent Error	3.31	3.34	3.34	2.76	2.79	2.60	
Average Difference (eV)	0.17	0.17	0.17	0.14	0.15	0.14	

investigated in the ABAB orientation that did not initially relax with a Z-component of the stress tensor below one kbar in magnitude. In the original study, all alternating monomers were rotated by 180 degrees, so in the ABAB orientation, all B monomers were rotated by 180 degrees. In this quick study, we found that by not rotating the tPA 180 degrees, we could relax the system to a point where the Z-component of the stress tensor was near zero, as shown in table S4. This relaxed polymer also did not crumple during this process, as shown in Fig. S3A.

Looking at the results in table S4, a conclusion can be drawn that if we were to continue decreasing the Z-component of the UC, the Z-component of the stress tensor would eventually reduce to a value less than the one kbar threshold. This would, however, lead to a crumpling of the SOP as shown in figure S3B, which is not experimentally observed. In most cases, we could see the start of the crumpling process in the SOP by observing how the Z-component of the stress tensor changed with respect to the Z-component of the UC. As the UC value decreased, the stress tensor value also reduced. Then, the stress tensor value would increase in magnitude at a specific UC value, followed by a continuous decrease. Looking at the results from the system that started with a zero degree rotation, this crumpling effect took

place as the UC was decreased from 5.721 Å down to 5.521 Å , as shown in the first row of table S4 and figure S3.

Table S4: Z-component of the stress tensor as a function of UC and rotation

	6.021 Å	5.921 Å	5.821 Å	5.721 Å	5.621 Å	5.521 Å
0	-3.27	-2.26	-1.20	-0.04	-1.32	-1.08
10	-3.24	-2.27	-1.24	-1.64	-1.31	-1.09
20	-3.33	-2.38	-1.97	-1.63	-1.38	-1.09
30	-3.48	-2.53	-1.89	-1.61	-1.36	-1.09
40	-3.29	-2.33	-1.98	-1.63	-1.36	-1.08
50	-3.30	-2.34	-1.97	-1.65	-1.36	-1.12
60	-3.29	-2.33	-1.30	-1.64	-1.36	-1.10
70	-3.31	-2.30	-1.28	-1.65	-1.34	-1.08
80	-3.33	-2.34	-1.95	-1.65	-1.34	-1.09
90	-3.28	-2.32	-1.31	-1.65	-1.34	-1.09
100	-3.37	-2.59	-1.93	-1.65	-1.34	-1.09
110	-3.50	-2.59	-1.98	-1.57	-1.32	-1.11
120	-3.31	-2.31	-1.26	-1.67	-1.32	-1.12
130	-3.30	-2.30	-1.26	-1.66	-1.33	-1.11
140	-3.29	-2.31	-1.28	-1.65	-1.31	-1.11
150	-3.28	-2.27	-1.21	-1.68	-1.32	-1.11
160	-3.29	-2.27	-1.21	-1.64	-1.33	-1.09
170	-3.44	-2.48	-1.89	-1.61	-1.36	-1.09
180	-3.24	-2.26	-1.21	-1.64	-1.32	-1.10

During the initial investigation, we repeated the rotation study for the PFU-PPY AABB-oriented SOP that failed to relax below the one kbar threshold to show that this starting angle dependency also carried through to the AABB orientation. In this case, the rotation angle was between the planes of both A monomers and B monomers, where the rotation between the first A and second A monomers was 180 degrees, and similarly for the B monomers. For this SOP, we found multiple starting angles that lead to a Z-component of the stress tensor less than the one kbar threshold, and their calculated values are presented in table S5. Except for the 10-degree starting angle, all calculated values were very similar. We also looked at the difference in energy between each system with respect to the zero-degree starting angle and found that there is very little difference, especially when compared to the total energy of the system, which is six orders of magnitude larger than these differences. This suggests that

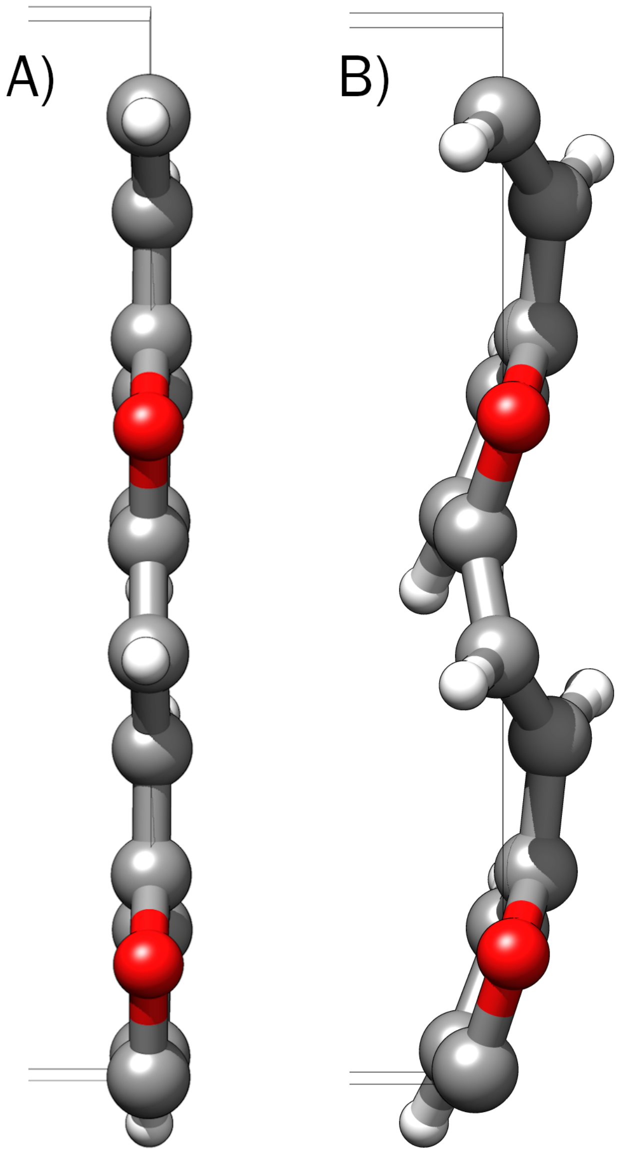

Figure S3: The PFU-tPA ABAB oriented polymer relaxed with a UC of A) 5.721 Å and B) 5.521 Å

when synthesized, the PFU-PPY AABB SOP has multiple stable orientations with similar electronic properties.

Table S5: Calculated values for the PFU-PPY AABB SOP with starting angles that lead to stress tensor values less than one kbar in magnitude.

	BG	HOCO	LUCO	Fermi	dE from 0-rot	Z-Pressure Å
0	1.53	-3.59	-2.06	-2.49	0.00	-0.79
10	1.59	-3.65	-2.06	-2.84	0.13	-0.87
20	1.53	-3.59	-2.06	-2.49	0.00	-0.78
70	1.53	-3.59	-2.06	-2.50	-0.02	-0.86
90	1.52	-3.58	-2.06	-2.49	-0.01	-0.81
100	1.52	-3.58	-2.06	-2.49	-0.01	-0.80
110	1.53	-3.58	-2.06	-2.49	-0.01	-0.82

Results for the SOP Mixtures

Tables S6 and S7 present the calculated BG and IP values of these mixtures and provide "side-by-side" comparisons when having different orientations. Some calculations resulted in unrealistic stresses that did not go below 1 kbar; we indicate those with NA.

Table S6: Corrected BG (eV) for a mixture a SCPs in the ABAB orientation (Red) and AABB orientation (Blue). NA denotes a calculation with stress above 1 kbar.

	tPA	PPP	PTH	PPY	PFU	P3MT	P3MP	P3MF
tPA	1.82	2.26	1.68	1.92	NA	1.71	1.89	NA
PPP	2.47	2.81	2.42	2.57	NA	2.24	NA	NA
PTH	1.74	2.89	1.99	2.28	NA	2.32	2.36	NA
PPY	1.99	3.20	2.30	2.76	2.56	2.49	NA	NA
PFU	1.69	3.25	2.13	2.56	2.38	NA	NA	NA
P3MT	2.13	3.35	2.12	2.39	2.20	2.03	2.26	NA
P3MP	2.39	3.22	2.40	2.78	2.55	2.28	2.75	NA
P3MF	3.03	3.31	2.27	2.60	2.42	2.10	2.52	2.37

Our main observation was the wide variety of new BGs and IPs that arose from mixing two SOPs and a sizable change in one value with little change in the other. A prime example is the mixture of tPA and PPY in the ABAB and AABB orientations, where the BGs were

Table S7: Corrected IP (eV) for a mixture a SCPs in the ABAB orientation (Red) and AABB orientation (Blue).

	tPA	PPP	PTH	PPY	PFU	P3MT	P3MP	P3MF
tPA	5.00	5.29	4.96	4.51	NA	4.81	4.34	NA
PPP	5.30	5.49	5.26	4.89	NA	5.24	NA	NA
PTH	4.82	5.45	5.00	4.75	NA	5.06	4.68	NA
PPY	4.42	5.16	4.72	4.49	4.57	4.72	NA	NA
PFU	4.59	5.45	4.89	4.59	4.77	NA	NA	NA
P3MT	5.01	5.57	4.94	4.63	4.78	4.74	4.43	NA
P3MP	4.57	5.04	4.66	3.73	4.44	4.40	3.57	NA
P3MF	5.47	5.37	4.83	4.43	4.60	4.53	4.25	4.37

1.99 eV and 1.92 eV, and the IPs were 4.42 eV and 4.51 eV, respectively. Looking at these values from the perspective of pure PPY, this mixture maintained the approximate IP while decreasing the BG by ~ 0.7 eV. Characterizing the BG and IP of a PTH and tPA mixture would, according to the results presented in Tables S6 and S7, be a good check of the validity of our calibrations. The two orientations of the monomers have relatively the same BG and IP values (0.06 eV for the BG and 0.14 eV for the IP).

References

(S1) KOVACIC, P.; JONES, M. B. ChemInform Abstract: Dehydro Coupling of Aromatic Nuclei by Catalyst-Oxidant Systems: Poly-(p-phenylene). *ChemInform* **1987**, *18*.

(S2) Chung, T.-C.; Kaufman, J. H.; Heeger, A. J.; Wudl, F. Charge storage in doped poly(thiophene): Optical and electrochemical studies. *Physical Review B* **1984**, *30*, 702–710.

(S3) Glenis, S.; Benz, M.; LeGoff, E.; Schindler, J. L.; Kannewurf, C. R.; Kanatzidis, M. G. Polyfuran: a new synthetic approach and electronic properties. *Journal of the American Chemical Society* **1993**, *115*, 12519–12525.

(S4) Pichler, K.; Halliday, D. A.; Bradley, D. D. C.; Burn, P. L.; Friend, R. H.;

Holmes, A. B. Optical spectroscopy of highly ordered poly(p-phenylene vinylene). *Journal of Physics: Condensed Matter* **1993**, *5*, 7155–7172.

(S5) Avilov, I.; Marsal, P.; Brédas, J.-L.; Beljonne, D. Quantum-Chemical Design of Host Materials for Full-Color Triplet Emission. *Advanced Materials* **2004**, *16*, 1624–1629.

(S6) Onoda, M.; Tada, K.; Shinkuma, A. In situ polymerization process of polypyrrole ultrathin films. *Thin Solid Films* **2006**, *499*, 61–72.

(S7) Brown, P. J.; Thomas, D. S.; Köhler, A.; Wilson, J. S.; Kim, J.-S.; Ramsdale, C. M.; Sirringhaus, H.; Friend, R. H. Effect of interchain interactions on the absorption and emission of poly(3-hexylthiophene). *Physical Review B* **2003**, *67*.

(S8) Groenendaal, L.; Jonas, F.; Freitag, D.; Pielartzik, H.; Reynolds, J. R. Poly(3,4-ethylenedioxythiophene) and Its Derivatives: Past Present, and Future. *Advanced Materials* **2000**, *12*, 481–494.

(S9) Lankinen, E.; Pohjakallio, M.; Sundholm, G.; Talonen, P.; Laitinen, T.; Saario, T. Application of in situ UV-vis spectroscopy and an in situ dc resistance measurement technique to the study of a poly(thiophene-3-methanol) film. *Journal of Electroanalytical Chemistry* **1997**, *437*, 167–174.

(S10) Moon, D. K.; Ezuka, M.; Maruyama, T.; Osakada, K.; Yamamoto, T. Kinetic study on chemical oxidation of leucoemeraldine base polyaniline to emeraldine base. *Macromolecules* **1993**, *26*, 364–369.

(S11) Sharma, H. S.; Park, S.-M. Electrochemistry of Conductive Polymers. *Journal of The Electrochemical Society* **2004**, *151*, E61.

(S12) Zhang, S.; Nie, G.; Han, X.; Xu, J.; Li, M.; Cai, T. Electrosyntheses of high quality free-standing poly(9-fluorenone) films in boron trifluoride diethyl etherate. *Electrochimica Acta* **2006**, *51*, 5738–5745.

(S13) Kamat, S. V.; Yadav, J.; Puri, V.; Puri, R.; Joo, O. Characterization of poly (3-methyl thiophene) thin films prepared by modified chemical bath deposition. *Applied Surface Science* **2011**, *258*, 482–488.

(S14) Moses, D.; Feldblum, A.; Ehrenfreund, E.; Heeger, A. J.; Chung, T. C.; MacDiarmid, A. G. Pressure dependence of the photoabsorption of polyacetylene. *Physical Review B* **1982**, *26*, 3361–3369.

(S15) Durani, S. M.; Khawaja, E. E.; Masoudi, H. M.; Bastl, Z.; Šubrt, J.; Galíková, A.; Pola, J. IR laser ablative desulfurization of poly(1,4-phenylene sulfide). *Journal of Analytical and Applied Pyrolysis* **2005**, *73*, 145–149.

(S16) Eckhardt, H.; Shacklette, L. W.; Jen, K. Y.; Elsenbaumer, R. L. The electronic and electrochemical properties of poly(phenylene vinylenes) and poly(thienylene vinylenes): An experimental and theoretical study. *The Journal of Chemical Physics* **1989**, *91*, 1303–1315.

(S17) Phillips, S.; Worland, R.; Yu, G.; Hagler, T.; Freedman, R.; Cao, Y.; Yoon, V.; Chiang, J.; Walker, W.; Heeger, A. Electroabsorption of polyacetylene. *Physical review B* **1989**, *40*, 9751.

(S18) Arjomandi, J.; Holze, R. Electrochemical preparation and in situ characterization of poly (3-methylpyrrole) and poly (3-methylpyrrole-cyclodextrin) films on gold electrodes. *Open Chemistry* **2008**, *6*, 199–207.

(S19) Palacios, R. E.; Barbara, P. F. Single molecule spectroscopy of poly 3-octyl-thiophene (P3OT). *Journal of fluorescence* **2007**, *17*, 749–757.

(S20) Berlin, A.; Pagani, G.; Zotti, G.; Schiavon, G. Electrochemical polymerization of 1H, 7H-pyrrolo [2', 3': 4, 5]-thieno [3, 2-b] pyrrole and 4H-dithieno [3, 2-b; 2', 3'-d] pyrrole. *Die Makromolekulare Chemie* **1992**, *193*, 399–409.

(S21) Brédas, J. L.; Chance, R. R.; Baughman, R. H.; Silbey, R. Ab initio effective Hamiltonian study of the electronic properties of conjugated polymers. *The Journal of Chemical Physics* **1982**, *76*, 3673–3678.

(S22) Bredas, J. L.; Silbey, R.; Boudreaux, D. S.; Chance, R. R. Chain-length dependence of electronic and electrochemical properties of conjugated systems: polyacetylene polyphenylene, polythiophene, and polypyrrole. *Journal of the American Chemical Society* **1983**, *105*, 6555–6559.

(S23) Brédas, J. L.; Elsenbaumer, R. L.; Chance, R. R.; Silbey, R. Electronic properties of sulfur containing conjugated polymers. *The Journal of Chemical Physics* **1983**, *78*, 5656–5662.

(S24) Barta, P.; Kugler, T.; Salaneck, W.; Monkman, A.; Libert, J.; Lazzaroni, R.; Brédas, J. Electornic structure of emeraldine and pernigraniline base: A joint theoretical and experimental study. *Synthetic Metals* **1998**, *93*, 83–87.

(S25) Micaroni, L.; Nart, F.; Hümmelgen, I. Considerations about the electrochemical estimation of the ionization potential of conducting polymers. *Journal of Solid State Electrochemistry* **2002**, *7*, 55–59.

(S26) Eakins, G. L.; Alford, J. S.; Tiegs, B. J.; Breyfogle, B. E.; Stearman, C. J. Tuning HOMO-LUMO levels: trends leading to the design of 9-fluorenone scaffolds with predictable electronic and optoelectronic properties. *Journal of Physical Organic Chemistry* **2011**, *24*, 1119–1128.

(S27) Guan, Z.-L.; Kim, J. B.; Wang, H.; Jaye, C.; Fischer, D. A.; Loo, Y.-L.; Kahn, A. Direct determination of the electronic structure of the poly(3-hexylthiophene):phenyl-[6,6]-C61 butyric acid methyl ester blend. *Organic Electronics* **2010**, *11*, 1779–1785.
